# Perianesthetic Management in a Patient With Alzheimer's Disease Complicated With Pulmonary Infection Who Underwent Emergency Right Artificial Femoral Head Replacement Surgery: A Case Report and Review of the Literature

**DOI:** 10.1155/crdi/4053129

**Published:** 2025-07-02

**Authors:** Wenying Song, Jing Huang, Ying Li, Jiajia Liu, Hui Ding

**Affiliations:** ^1^Department of Anesthesiology, Shaanxi Provincial Hospital, Xi'an 710068, China; ^2^Xi'an Medical University, Xi'an 710068, China

**Keywords:** case report, elderly patients, fracture, preoperative evaluation

## Abstract

**Background:** Choosing the appropriate timing for surgery is a common clinical problem when hip fracture occurs in elderly patients with a variety of complications.

**Case Presentation:** We present a fatal case involving an 84-year-old male patient with bradycardia who underwent cardiac pacemaker implantation and presented with cognitive impairment, Alzheimer's disease, and pulmonary infection and had an American Society of Anesthesiologists (ASA) IV status; this patient underwent emergency right artificial femoral head replacement surgery. We strengthened our perioperative evaluation and monitoring, but the patient's condition did not improve after the operation.

**Conclusion:** We utilized multidisciplinary consultation before the operation, attempted to improve the state of the patient before the operation, chose an appropriate anesthetic scheme during the operation, strengthened cardiopulmonary protections, and returned the patient to the ward safely after the operation; however, the patient did not recover well post operation.

## 1. Background

Surgery is the main method of treatment for senile femoral head fractures; however, elderly patients often simultaneously experience a variety of diseases due to a decline in physiological function and a decrease in the reserve capacity of whole-body organs. The ability of the body to regulate all types of stressors during operation and anesthesia decreases. Therefore, ensuring the perioperative safety of these patients is a common problem in the clinical work of anesthesiologists.

## 2. Case Presentation

### 2.1. Summary of Medical History

1. An 84-year-old Chinese male with a BMI of 13.1 kg/m^2^ was admitted to the hospital because of limited movement of the right lower limb for 2 days following a trauma. The patient had an unsteady gait and accidentally fell at home 2 days prior. At that time, there was no loss of consciousness; no symptoms such as headache, nausea, or vomiting; limited activity; no relief of limb pain; and limited activity after bed rest. The patient visited the Department of Neurology of our hospital. Combined with the patient's medical history, physical examination, and x-ray examination, the patient was transferred to orthopedics for further treatment and was prepared for femoral head replacement.
2. Past history: the patient presented with bradycardia for 9 years and experienced cognitive impairment after undergoing cardiac pacemaker implantation in 2018. He had a 1-year history of Alzheimer's disease, and he had been regularly taking donepezil, but the dosage details are unknown. He denied any history of food or drug allergies.
3. Admission diagnosis: Alzheimer's disease, right femoral neck fracture (subcephalic type), cardiac pacemaker operation, pulmonary infection, anemia, hypoproteinemia, hypocalcemia, and subclinical hypothyroidism. Brief information of the patient is shown in Table 1.
4. Physical examination: his BP and HR were 120/70 mmHg and 78 beats/min, respectively, with a respiratory rate (RR) of 19 breaths/min, a body temperature of 36.8°C, and an SpO_2_ of 92% (nasal catheter 2 L/min). Auscultation indicated sputum sounds in both lungs. The patient was apathy, in the supine position, was unresponsive to voice, and his activity was limited.
5. Laboratory and auxiliary examination: for detailed results of patient examinations, see Table 2.
  An electrocardiogram (ECG) revealed that the sinus heart rate, ventricular extrasystole, atrial pacing, and pacemaker function were normal. An echocardiogram revealed good myocardial contractility with a normal ejection fraction, a pacemaker implanted with no obvious abnormalities in the pacemaker guide wire position and echo, and a small amount of pericardial effusion. A chest CT revealed a scattered infection in both lungs, which was reexamined after treatment; changes after cardiac pacemaker implantation; and signs of aorta and coronary artery sclerosis. A craniocerebral CT revealed demyelination of the bilateral paraventricular white matter with multiple luminal infarcts and simultaneous brain atrophy. Bilateral hip joint CT revealed a fracture of the right femoral neck with swelling of the surrounding soft tissue.
6. Before the operation, we invited relevant departments to consult.
  Respiratory doctors noted that the patient had a cough, expectoration, a thick lung breathing sound accompanied by a phlegm sound, and a water-drinking cough phenomenon; thus, anti-infective expectorant treatment was recommended. Cardiologists believed that the perioperative cardiovascular accident risk was higher because the patient was elderly and was complicated with pulmonary infection, anemia, hypoproteinemia, and troponin elevation.
7. Traditional Chinese medicine (TCM) technology: the acupoint application treatment involved Huoxue application at both bilateral Zusanli and Sanyinjiao points, once a day, for 4–6 h and Leihuo application at both the Zusanli and Xuehai points once a day for 30 min.
8. Preoperative evaluation: the patient was classified as ASA IV status with an estimated perioperative mortality of 7.8%–23%, a metabolic equivalent < 1 MET, and an Assess Respiratory Risk in Surgical Patients in Catalonia score of 49 [1], which represents a high risk (> 44). The incidence of postoperative pulmonary complications (PPCs) was estimated to be as high as 42.1%. The safety Caprini [2] rating scale predicted a very high risk of deep venous thrombosis (> 9). The predicted incidence of deep venous thrombosis was 40%–80%. The predicted risk of mortality ranged from 1% to 5%. The Nottingham hip fracture score was 7, and the predicted 30-day mortality was 23%.

### 2.2. Anesthesia and Surgery

1. Preoperative evaluation: a medication evaluation indicated the need for continued Alzheimer's disease medication up to 1 day before the operation [3], and steps were taken to prevent deep venous thrombosis by the subcutaneous injection of low-molecular-weight heparin sodium (2500 IU) that was discontinued > 12 h before the operation.
2. Mode of anesthesia: the results of two blood coagulation function tests revealed abnormal blood coagulation function, but thromboelastography suggested high platelet function. Based on the patient's age, pulmonary function, and family members' opinions, the use of a right iliac fascia block plus lumbar–epidural anesthesia to prepare for general anesthesia was proposed [4].
3. Preanesthesia treatment: after the patient entered the OR, the patient was not conscious and had no speech response. After tripartite verification, a venous pathway of the right upper limb was established with a 16G indwelling needle. His measurements were as follows: BP, 120/66 mmHg; ECG (HR of 70 bpm, occasional ventricular extrasystole); SpO_2_, 91%; body temperature, 36.0°C; and BIS, 95. A right radial artery puncture tube was used to maintain local anesthesia with continuous arterial blood pressure monitoring measuring 130/75 mmHg. The patient received cardiac medicine and a heating blanket to keep warm.
4. Anesthesia: the anesthesia was administered as follows: ultrasound-guided right iliac fascia block (0.33% ropivacaine, 30 mL), combined spinal and epidural anesthesia at L3-4, subarachnoid injection of a mild specific gravity drug (0.5% ropivacaine (8 mg) and aseptic injection water diluted to 2 mL), and epidural catheterization. There was no need to change the patient's position, and the anesthesia level was T10 after 5 min; then, the operation began.
5. Anesthesia maintenance: anesthesia was maintained by the intraoperative intravenous infusion of dexmedetomidine (0.4 μg/kg/h), nasal catheter oxygen inhalation (5 L/min), and the intraoperative preparation of m-hydroxylamine and norepinephrine to maintain an MAP > 70 mmHg.
6. Intraoperative management: we used antibiotics during the operation and shortened the operation time. There was obvious blood oozing from the skin incision, and plasma was infused.
7. Postoperative analgesia: for the epidural analgesia, 1 mg of morphine was administered 20 min before the end of the operation, and an intravenous analgesia pump was used after the operation that contained 50 μg of sufentanil, 16 mg of ondansetron, 100 mL of normal saline, 0.5 mL of background infusion, and 0.5 mL of PCA and had a locking time of 15 min.
8. Input and output: the input volume was 900 mL (500 mL of crystal solution and 400 mL of plasma), and the output volume was 700 mL (bleeding volume of 300 mL and urine volume of 400 mL).

### 2.3. Postoperative Management and Prognosis

1. Postoperative management: the operation lasted 75 min, and the patient was awake after the operation and was sent to the PACU for observation for 1 h. The vital signs of the patient were stable, and the patient returned to the ward safely.
2. Prognosis: on the first day after the operation, the patient was more conscious than before but still had excessive sleep and responded incoherently. His HR was 60 bpm, his blood pressure was stable, his SpO_2_ was 96% (nasal catheter oxygen inhalation 2 L/min), his NRS score was 3 points, his cough and expectoration had worsened, and aerosol inhalation and antibiotic treatment were continued. On the fourth day after the operation, the patient entered a state of lethargy and was coughing up sputum with obvious wet rales in both lungs. His SpO_2_ decreased slowly from 92% to 90% (with nasal catheter inhalation of 2–5 L/min of oxygen), his HR increased from 80 to 100 bpm, and his urine volume decreased. In addition to receiving atomization expectoration as an anti-infective treatment, many bedside photos revealed exudative lesions of both lungs, and cardiology and respiratory department consultation suggested that the patient remain in the ICU to receive tracheal intubation ventilator treatment. Family members refused and signed the patient out of the hospital.

## 3. Discussion

### 3.1. Preoperative Evaluation and Preparation

In elderly patients with obvious osteoporosis, minor trauma can cause fractures, and these patients are prone to serious complications. At present, the main clinical treatment is surgery. According to the existing surgical guidelines, early surgery is preferred, but elderly patients often have multiple underlying diseases simultaneously, and the risks of operation and anesthesia are relatively increased, which affects the outcome of patients after surgery. Thus, good preoperative evaluation and preparation are particularly important. The bleeding and clotting time of the patient in this study was prolonged twice before the operation, which may have been due to the use of oral anticoagulants. The presence of liver function damage or any other disease should be evaluated before surgery. In this case, a review of the patient's relevant medical history revealed that the psychiatry department used low-molecular-weight heparin sodium to prevent thrombosis and the orthopedics department had used it routinely before the operation; the drug administration was stopped 12 h before the operation. It has been reported in the literature that the preoperative subcutaneous injection of low-molecular-weight heparin and unfractionated heparin is convenient and safe, with few severe bleeding complications, and that there is generally no need for the routine detection of coagulation function changes [5]. Therefore, the patient was treated with a subcutaneous injection of low-molecular-weight heparin sodium (2500 IU) that was stopped more than 12 h before the operation to prevent deep venous thrombosis.

### 3.2. Perioperative Lung Protection

The purpose of preoperative lung assessment is to minimize PPCs. PPCs can increase early postoperative morbidity and mortality, prolong hospitalization and time in the ICU, and increase the rate of readmission. The Assess Respiratory Risk in Surgical Patients in Catalonia was used to assess the preoperative lung status of this patient. The patient's age was > 80 years (risk score of 16), the preoperative SpO_2_ was 91%∼95% (risk score of 8), the risk score of respiratory tract infection in the past month was 17, and the total score was 49, which corresponded to a high risk of PPCs, with a predicted incidence of 42.1%. Therefore, the implementation of perioperative lung protection strategies and the effective prevention and treatment of PPCs has important clinical significance for improving the survival rate and accelerating the rehabilitation of patients. Preoperative preventive measures include quitting smoking after admission, performing preoperative respiratory muscle exercises, and administering perioperative aerosol inhalation preventive medication [6]. Intraoperative preventive measures include the intraoperative prophylactic use of antibiotics, shortening the operation time and implementing an intraoperative protective lung ventilation strategy and restricted infusion. Postoperative preventive measures include postoperative analgesia, respiratory physiotherapy, and early out-of-bed activities.

Because this patient could not communicate, we could only use preventive drugs before the operation; there was no mechanical ventilation during the operation, so the postoperative lung protection strategy was particularly important. Effective postoperative analgesia plays an important role in postoperative lung protection strategies because postoperative pain can induce pain-related atelectasis and a lack of lung ventilation; thus, good postoperative analgesia and the use of anesthetic drugs with short half-lives can help reduce the risk of PPCs [7]. To improve the effectiveness of postoperative analgesia, a right iliofascial block was performed before the operation. Morphine was injected into the epidural space 20 min before the operation ended, and PCIA was administered after the operation. The patient had Alzheimer's disease and was prone to aspiration, decreased cough ability, and decreased lung self-purification ability. To prevent pulmonary infection, avoiding reaspiration through strengthening oral care, providing a perioperative gastric tube and nasal feeding nutrition, and implementing anti-infection treatment are necessary. A key limitation is that some patients are unable to participate in postoperative respiratory physiotherapy programs, such as exercise respiratory muscle functions, including deep breathing and cough. Such patients may only be able to be patted on the chest with the help of nurses and family members; however, these methods are also limited for deep sputum excretion. Furthermore, some patients cannot get out of bed, which is also an important reason for increased risk of postoperative pulmonary infection [8]. His BMI was 13.8 kg/m^2^, and his malnutrition and abilities to handle infection and surgical stress all worsened.

### 3.3. Perioperative Brain Protection

This patient had a history of “postoperative cognitive dysfunction” and subsequently suffered from Alzheimer's disease, coupled with a decline in physiological function, fluctuations in intraoperative hemodynamics, and the effects of inflammatory reactions and oxidative stress caused by surgical stress. The risk of perioperative cognitive dysfunction increases and subsequently affects postoperative recovery [9], so strengthening perioperative brain protection is particularly important.

Drugs for the treatment of Alzheimer's disease can be used until 1 day prior to the operation, and we should continue to give attention to preventing infection, correcting ion disorders, preventing low perfusion ischemia, avoiding hypocapnia, hyperglycemia, or hypoglycemia, and providing anti-inflammatory and antistress treatment. Vahedi et al. reported that dexmedetomidine could reduce the level of brain metabolism, reduce oxygen consumption, protect nerve cells, improve parasympathetic nerve activity, anti-inflammatory effects, and reduce central inflammatory reactions [10]. Dexmedetomidine has a certain neuroprotective effect on elderly patients undergoing orthopedic surgery [11] and can significantly reduce the incidence of postoperative delirium in elderly patients [12]. Therefore, in this patient, dexmetomidine was injected intravenously during anesthesia maintenance, and load infusion was not performed considering the side effect that dexmetomidine could slow HR. Second, Zhang et al. reported that the cognitive function score of elderly patients in an intraspinal anesthesia group was greater than that in a general anesthesia group during hip arthroplasty [13], so we chose intraspinal anesthesia for this patient to improve postoperative brain function and reduce postoperative complications. However, the patient underwent an emergency operation; thus, the preparation for examination and intraoperative monitoring were insufficient.

### 3.4. TCM

TCM has been applied for the inhibition and treatment of numerous diseases and has an irreplaceable role in recovery and healthcare. In the classical theory of Chinese acupuncture, the Zusanli and Sanyinjiao acupoints are the first choices for Huoxue application, with the Zusanli and Xuehai points utilized for the Leihuo technique. However, there is still a lack of evidence on appropriate and optimal acupuncture interventions. TCM pays attention to meridians, the theories are quite abstract, and some of them need to be verified or explained with the help of Englisch medicine. The theories of Englisch medicine are relatively wide and easy to understand. Therefore, the two can be combined to play a better role.

## Figures and Tables

**Table 1:** Information of the patient.

Age	Gender	Smoking status	Family history	Other comorbidities
84 years	Male	No	No	Alzheimer's disease, cardiac pacemaker operation, pulmonary infection, anemia, hypoproteinemia, hypocalcemia, subclinical hypothyroidism

**Table 2:** Laboratory examination.

Routine blood parameters	RBC	WBC	Hb	Hct	Plt
	3.14 × 10^9^/L	5.05 × 10^9^/L	94 g/L	0.29	188 × 10^9^/L
Blood biochemistry	ALT	AST	BUN	Cr	UA
	17 U/L	45 U/L	6.94 mmol/L	16.61 μmol/L	71.5 μmol/L
Electrolyte	K^+^	Ca^2+^			
	4.0 mmol/L	2.03 mmol			
Protein	TP	ALB			
	56.3 g	28.1 g/L			
Myocardial injury markers	CK-MB	Myoglobin	Hs-TnT	NT-proBNP	
	5.8 ng/m	280 ng/mL	0.024 ng/m	469 pg/mL	

*Blood clotting function*					
First	PT	APTT	Fib	FDP	D-2 polymer
	14.5 s	39.9 s	6.92 g/L	6.6 mg/L	1.68 mg/L
2 days later	PT	APTT	Fib	FDP	D-2 polymer
	13.9 s	54.9 s	6.92 g/L	6.6 mg/L	1.68 mg/L
Thromboelastography	R	*κ*	Angel	MA	CI
	5.8	1	74.2	71.6	2.4
Blood gas analysis	PH	PaO_2_	PaCO_2_	HCO_3_^−^	BE
	7.42	76 mmHg	38.8 mmHg	25.3 mmol/L	1 mmol/L

## Data Availability

The data that support the findings of this study are available from the corresponding author upon reasonable request.
